# Transcriptomic responses of hypothalamus to acute exercise in type 2 diabetic Goto-Kakizaki rats

**DOI:** 10.7717/peerj.7743

**Published:** 2019-09-24

**Authors:** Shuying Fu, Yuhuan Meng, Shudai Lin, Wenlu Zhang, Yuting He, Lizhen Huang, Hongli Du

**Affiliations:** School of Biology and Biological Engineering, South China University of Technology, Guangzhou, China

**Keywords:** Acute exercise, Type 2 diabetes (T2D), Hypothalamus, Goto-Kakizaki (GK) rat, RNA-Seq, Transcriptome

## Abstract

The hypothalamus has an integral role in energy homeostasis regulation, and its dysfunctions lead to the development of type 2 diabetes (T2D). Physical activity positively affects the prevention and treatment of T2D. However, there is not much information on the adaptive mechanisms of the hypothalamus. In this study, RNA sequencing was used to determine how acute exercise affects hypothalamic transcriptome from both type 2 diabetic Goto-Kakizaki (GK) and control Wistar rats with or without a single session of running (15 m/min for 60 min). Through pairwise comparisons, we identified 957 differentially expressed genes (DEGs), of which 726, 197, and 98 genes were found between GK and Wistar, exercised GK and GK, and exercised Wistar and Wistar, respectively. The results of Gene Ontology and Kyoto Encyclopedia of Genes and Genomes pathway enrichment revealed that lipid metabolism-related terms and pathways were enriched in GK and exercised GK rats, and nervous system related terms and pathways were enriched in exercised GK and Wistar rats. Furthermore, 45 DEGs were associated with T2D and related phenotypes according to the annotations in the Rat Genome Database. Among these 45 DEGs, several genes (*Plin2*, *Cd36*, *Lpl*, *Wfs1*, *Cck*) related to lipid metabolism or the nervous system are associated with the exercise-induced benefits in the hypothalamus of GK rats. Our findings might assist in identifying potential therapeutic targets for T2D prevention and treatment.

## Introduction

Diabetes is a global public health problem that is growing each year, resulting in considerable medical care costs and millions of deaths. It was estimated that in 2017, 424.9 million people worldwide suffered from diabetes, and nearly 87% to 91% of these were affected with type 2 diabetes (T2D) characterized by high blood glucose, and insulin resistance at an early stage (Ogurtsova et al., 2017). In Asian populations, the prevalence of T2D has increased rapidly in a short period. More than 60% of T2D patients came from Asia, of which nearly 114.4 million resided in China. However, compared with western counterparts, Asian T2D patients may have different patterns, especially the individuals with comparatively low mean body mass index (Yang & Weng, 2014; Ramachandran, Ma & Snehalatha, 2010). This clinical characteristic should not be ignored, and appropriate animal models are necessary to investigate the pathophysiology and treatments of T2D without obesity.

The Goto-Kakizaki (GK) rat, a T2D animal model which is spontaneous and non-obese, was generated by repeated inbreeding of Wistar rats which were selected at the upper limit of the normal distribution for glucose tolerance (Portha et al., 2012). Previous studies reported that the body weight of GK rats was lower than that of Wistar rats and GK rats also displayed glucose intolerance, hyperglycemia, and insulin resistance at the age of 8 weeks (Movassat et al., 2008; Nie et al., 2011). Considering these characteristics, GK rats may be helpful in studying pathogenic mechanisms and therapeutic approaches of human non-obese T2D patients.

The hypothalamus plays a vital role in regulating the energy homeostasis by integrating and adapting to peripheral signals, including nutrients (glucose and fatty acids), and hormones (insulin, leptin, ghrelin, etc.) (Lopez et al., 2016; Picard et al., 2014). Research has shown that hypothalamic dysfunctions contribute to the development of chronic conditions, such as T2D (Jais & Bruning, 2017; Chowen et al., 2016). We previously detected the disorder of transcriptome in the hypothalamus through RNA sequencing (RNA-Seq) analysis, which might be associated with pathogenesis in diabetic GK rats (Meng et al., 2016). Physical activity is a critical element in the management and prevention of T2D by improving blood glucose and insulin action (De Feo & Schwarz, 2013). It has been observed that only a single session of exercise can attenuate hyperglycemia and enhance insulin sensitivity (Colberg et al., 2010). Over the decades, many studies on exercise have focused on peripheral tissues to explore the adaptive mechanisms in the T2D (Sylow et al., 2017; Hussey et al., 2011; Fu et al., 2019). Interestingly, recent research suggested that exercise training of obese mice could improve high-fat diet impaired proopiomelanocortin (POMC)-expressing neurons, protect against inflammation and aid in maintaining energy balance in the hypothalamus (Ropelle et al., 2010; Laing et al., 2016). However, the effects of exercise on the hypothalamus remain unclear in T2D condition.

Here, we employed the RNA-Seq technology to assess the effects of acute exercise on hypothalamic transcriptome from 8 weeks old GK as well as Wistar rats with or without a single running session. We analyzed the differentially expressed genes (DEGs), and their functional associations. The results obtained may aid in better understanding of the role hypothalamus plays in the pathogenesis of T2D, extend our knowledge of the adaptive mechanisms of the hypothalamus in response to exercise and help identify potential therapeutic targets for T2D prevention and treatment.

## Material and Methods

### Ethical approval

The institutional review board, Guangdong Key Laboratory of Laboratory Animals, approved this study (Ethics certificate: IACUC2014029). Each experimental procedure was performed as per recommendations found in the Guide for the Care and Use of Laboratory Animals.

### Animals and exercise protocols

The SLAC Laboratory Animal Co., Ltd. (Shanghai, China) provided us with 6-week-old male GK rats (*n* = 20) and Wistar rats (*n* = 20). Female rats were excluded to avoid considering the effects of hormonal status (Jiang et al., 2015; Accattato et al., 2017). All GK and Wistar rats were housed in a specific pathogen-free animal room with relative humidity at 50∼60%, at 23∼24 °C, and a 12-h dark/light cycle. The water and standard rat chow were made accessible ad libitum to the animals, and they were weighed once a week.

After a week of adaptive feeding, all rats were made familiar to the exercise on a treadmill by a paradigm of progressive running on a motorized rodent treadmill (DSPT-202, Duanshi Manufacturing Plant, China) when they were 7 weeks old. On the first day, rats were acclimated to the treadmill equipment for 5 min at 8 m/min speed and then adapted for 10 min at 10 m/min. On the second and the third days, rats ran for 5 min at 10 m/min, and then at 15 m/min for 10 min, respectively. Acclimated rats were rested for 2 days and randomly assigned to an exercised, or a sedentary group (Wistar, exercised Wistar, GK, and exercised GK, *n* = 10/group).

We carried out the formal experiment when the rats were of 8 weeks age. The exercised Wistar and exercised GK rats underwent a single session of running on the treadmill at 15 m/min for 60 min, at approximately 60% of their VO_2max_ (Tsutsumi et al., 2015; Bedford et al., 1979; Scopel et al., 2006). Continuous running was encouraged by noise (Caron, Marqueste & Decherchi, 2016). For both exercised and sedentary groups, food and water were removed during the exercise session.

### Blood and tissue sampling

Blood and hypothalamus samples were harvested 2 h after exercise (Pourteymour et al., 2017; Takimoto & Hamada, 2014). Rats were administered anesthesia with pentobarbital sodium (intraperitoneal, 50 mg/kg body weight), then euthanized by abdominal aortic exsanguination. The samples of blood were collected in EDTA microcentrifuge tubes and centrifuged at 2000 × g at 4 °C for 15 min to separate plasma. The whole hypothalamus was dissected (defined rostrally by the optic chiasm, caudally by the mamillary body, ventrally by the anterior commissure, and laterally by the tuber cinereum and mamillary body complexes) (Houlahan et al., 2015; Borg et al., 2014), snap-frozen in liquid nitrogen, and stored at −80 °C until future study. For sedentary controls, rats from both Wistar and GK groups were anesthetized and euthanized at times corresponding to the exercised groups.

### Assessment of plasma glucose and insulin levels

Plasma glucose levels were determined through GLU-PIII slides using an automatic analyzer DRI-CHEM 7000i (both from Fujifilm, Japan). Levels of insulin were measured in triplicate using ELISA kits for Rat Insulin (Thermo Scientific, USA) with a plate reader SpectraMax M5 (Molecular Devices, USA) at 450 nm and 550 nm. Assays were directed as per instructions without modifications.

### Calculation of HOMA-IR indexes

An individual Homeostatic Model Assessment for Insulin Resistance (HOMA-IR) index was determined using the values of plasma glucose and insulin of each sample as per the formula: HOMA-IR = glucose (mmol/L) × insulin (µIU/ml)/22.5 (Matthews et al., 1985).

### RNA extraction, library preparation, and sequencing

In this study, six samples of hypothalamus from each group were used for RNA-Seq. TRIzol reagent (Thermo Scientific, Waltham, MA, USA) was used to extract total RNA, as per manufacturer’s recommendations. The RNA pellet was dried after giving 75% ethanol wash and resuspended in ultrapure nuclease-free water. The RNA degradation and contamination were detected by 1% formaldehyde-agarose gel electrophoresis. Then, the purity of RNA was assessed using the Qubit^^®^^ 3.0 Fluorometer (Life Technologies, CA, USA), and its integrity and concentration were determined by the RNA Nano 6000 Assay Kit of the Bioanalyzer 2100 system (Agilent Technologies, Santa Clara, CA, USA). Preparation of RNA-Seq libraries was done by RNA Library Prep Kit for Illumina^®^ (NEB, Ipswich, MA, USA) as per the manufacturer’s recommendations. Briefly, mRNA was extracted from total RNA by magnetic beads with attached poly-T oligos and fragmented using divalent cations in First Strand Synthesis Reaction Buffer (NEB, Ipswich, MA, USA). Synthesis of the first strand cDNA was done by addition of random hexamer primers, and second strand cDNA was subsequently performed using DNA polymerase I and RNase H. The QIAquick PCR Purification kit (Qiagen, Hilden, Germany) was used to purify cDNA. After terminal repair, adenylation of the 3′ ends and adapters added, target products were selected and amplified to construct the sequencing library which was qualified with the Qubit^®^ 3.0 Fluorometer. Finally, the Illumina HiSeq X platform (Illumina, San Diego, CA, USA) was used for sequencing and generating 150 bp paired-end reads.

### Quality control

To generate cleaned data, raw data in FASTQ format were processed using the following filtering criteria: (1) reads with quality less than 20 and a filter length over 50%; (2) reads with unidentified bases more than 5%; (3) reads with overrepresented adaptors; (4) low-quality base from 3’ end with a mass threshold of 10. For downstream analysis, the high-quality clean data were used.

### Differential gene expression analysis

The quality control passed reads were then aligned to the reference genome of *Rattus norvegicus* (Rnor_6.0, version 86) from Ensembl using STAR2 software (version 020201) (Dobin et al., 2013). The alignment ratios of samples were presented in Table S1 . To assemble the transcripts, StringTie (version 1.3.0) was used (Pertea et al., 2015). Additionally, a StringTie enabled Python script, prepDE.py was used to extract information on the read count and build count matrices. The edgeR package was used to analyze the differential gene expression (Robinson & Oshlack, 2010). The *p*-values were adjusted by applying the Benjamin-Hochberg method to control the false discovery rate. Genes were considered as expressed differentially if corrected *p*-value < 0.05.

### Functional enrichment analysis

Gene Ontology (GO) and Kyoto Encyclopedia of Genes and Genomes (KEGG) pathway enrichment analysis of DEGs were carried out using the Cytoscape (version 3.5.1) plug-in ClueGO (version 2.5.3) (Bindea et al., 2009). The KEGG pathways and GO terms were considered significantly enriched with an adjusted *p*-value < 0.05. The ClueGO was also used for clustering and visualizing GO terms to create functionally organized networks with default settings.

### Validation through quantitative Real-time PCR (qRT-PCR)

To confirm the accuracy and reproducibility of the RNA-Seq results, several DEGs associated with T2D or exercise were validated with qRT-PCR using the sequenced samples (Cao et al., 2018; Kim, Kim & Son, 2018; Ge et al., 2018). Total RNA was converted to cDNA using the Primescript™ RT reagent Kit with gDNA Eraser (Takara, Japan). The SYBR^®^ Premix Ex Taq™ II Kit (Takara, Japan) was used to prepare the qRT-PCR reaction mixture following the manufacturer’s instructions. Reactions were performed in the Light Cycler^^®^^ 96 Real-Time PCR system (Roche, Basel, Switzerland) with amplification procedure: 95 °C for 30 s, 45 cycles of 10 s at 95 °C and 60 °C for 30 s. The internal reference used was *Actb*, a housekeeping gene to normalize the expression levels of selected genes, and the ratios of relative expression were analyzed using the 2^−ΔΔCt^ method. Specific primers were designed by Primer Express (version 6.0) and presented in Table S2.

### Statistics

The results for physiological indexes and qRT-PCR analysis are presented as the mean ± standard error of the mean (SEM). Analyses of differences among groups were done by one-way analysis of variance (ANOVA) with Tukey’s *post hoc* analysis. The software GraphPad Prism 7.0 was used for statistical analyses. A significant level had *p*-value < 0.05.

## Results

### Animal characteristics

The characteristics of four groups (Wistar, exercised Wistar, GK, and exercised GK) were presented in Table 1. The body weights of all rats were measured before the study. At 8 weeks of age, Wistar rats were slightly heavier than GK rats, but there was no significant difference in body weights between Wistar and exercised Wistar, GK and exercised GK rats. A significantly higher level of random plasma glucose was observed in GK rats than in Wistar rats, and the insulin level was also elevated. After a single session of running, the level of plasma glucose was reduced in exercised GK rats in comparison with that of GK rats, indicating that acute exercise could alleviate hyperglycemia. Furthermore, the insulin level in exercised GK rats showed a decrease compared with that in GK rats. In contrast to exercised GK rats, there was no significant difference in plasma glucose and insulin levels between the two Wistar groups. To assess insulin resistance, apparent HOMA-IR indexes were calculated by the values of non-fasted plasma glucose and insulin (Nie et al., 2011). A significantly higher HOMA-IR index was observed in the GK rats than the other groups, indicating severe insulin resistance. The HOMA-IR index of exercised GK rats showed a distinct decrease in comparison with that of GK rats, indicating that insulin sensitivity was improved after acute exercise.

**Table 1:** Physiological parameters of Wistar and GK rats with or without acute exercise.

**Group**	**Body weight (g)**	Plasma glucose (mmol/L)	**Insulin (pmol/L)**	**HOMA-IR index**
W	274.8 ± 2.9	7.0 ± 0.11	311.5 ± 75.66	14.36 ± 3.7
EW	277.7 ± 2.0	7.15 ± 0.15	257 ± 31.31	14.27 ± 2.87
GK	261.1 ± 2.9^*^	17.93 ± 1.26^**^	814.6 ± 97.16^**^	91.9 ± 9.6^**^
EGK	260.6 ± 4.6	14.69 ± 0.76^†^	398.2 ± 47.17^‡^	38.16 ± 5.0^‡^

**Notes.**

W Wistar group EW exercised Wistar group GK GK group EGK exercised GK group

Analyses of data were done by ANOVA, and differences between groups were identified by Tukey’s *post hoc* analysis.

Values are mean ± SEM; n = 10/group.

** *p* < 0.01 vs. W.

* *p* < 0.05 vs. W.

‡ *p* < 0.01 vs. GK.

† *p* < 0.05 vs. GK.

### Identification of DEGs

**Figure 1:**
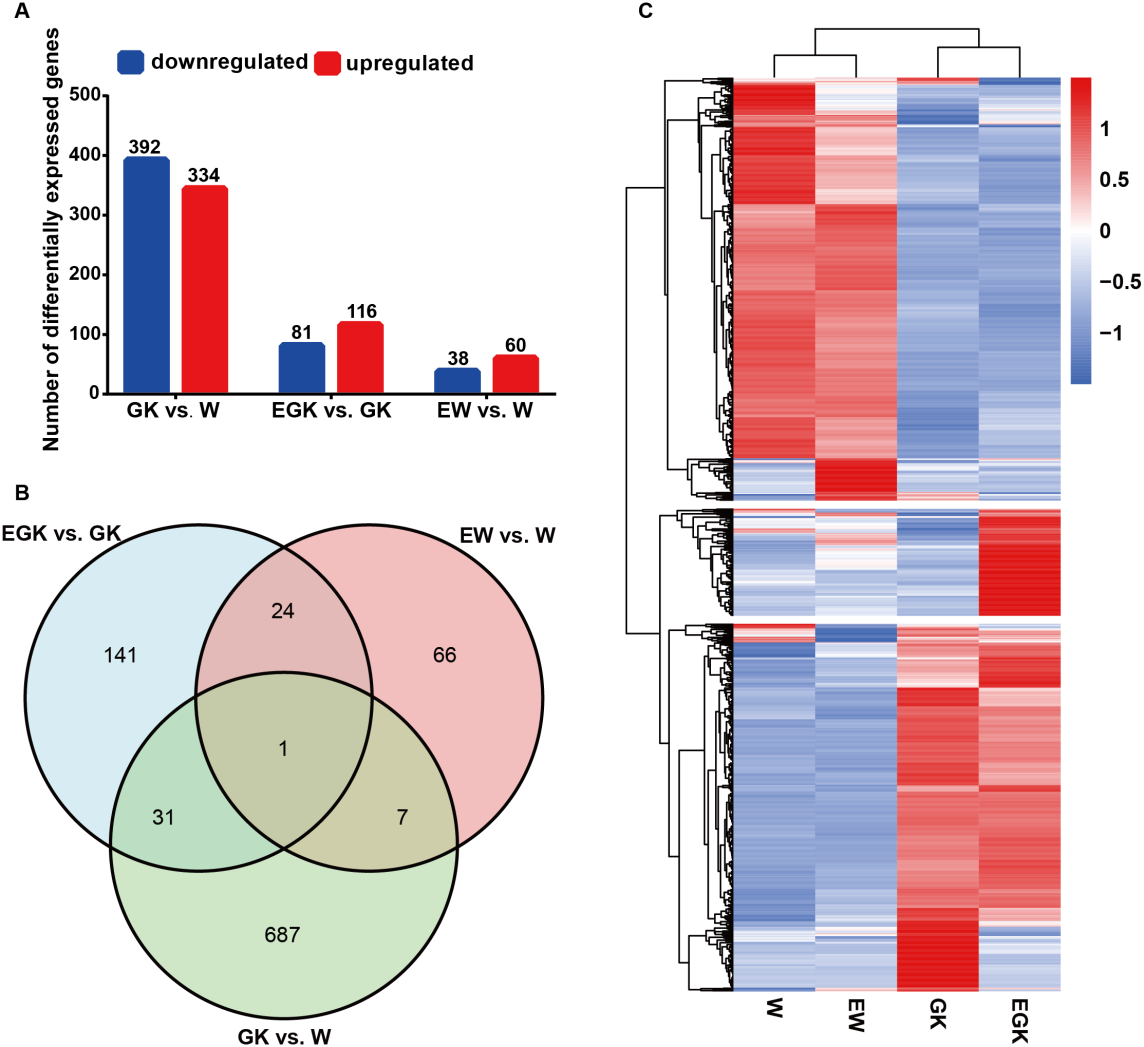
Changes in hypothalamic gene expression profiles. (A) Numbers of upregulated and downregulated DEGs in the three comparisons (GK vs. W, EGK vs. GK, and EW vs. W). (B) The overlap of DEGs in the three comparisons presented as a Venn diagram. (C) Heatmap representing the relative expression levels of DEGs in the four groups (W, Wistar group; EW, exercised Wistar group; GK, GK group; EGK, exercised GK group).

To investigate the regulatory mechanisms involved in the adaptive responses to acute exercise, we profiled the hypothalamus transcriptome in four groups. We identified 957 DEGs in the three comparisons of GK vs. Wistar, exercised GK vs. GK, and exercised Wistar vs. Wistar, and their details were listed in Table S3. Among the identified DEGs, 726 genes were found between rats of GK and Wistar groups, with 392 downregulated genes and 334 upregulated genes (Fig. 1A). A single session of running resulted in the differential expression of 197 genes in the exercised GK rats in comparison with the sedentary controls, of which 81 genes were downregulated, and 116 genes were upregulated (Fig. 1A). We observed 98 genes with a significantly altered expression in the exercised Wistar rats compared with Wistar rats. Among these genes, 38 were downregulated, and 60 genes were upregulated (Fig. 1A). Analysis using Venn diagram illustrated that only one DEG (*Fosb*) was shared in the mentioned three comparisons (Fig. 1B). Besides, the number of overlapping DEGs between GK vs. Wistar and exercised GK vs. GK, exercised GK vs. GK and exercised Wistar vs. Wistar was 32, 25, respectively. We then analyzed the expression patterns of all DEGs using hierarchical clustering and presented them in a heatmap (Fig. 1C). These genes were clustered into 3 major groups (>100 genes/group). In group 1, many genes had elevated expression levels in sedentary and exercised Wistar rats. The genes in group 3 exhibited elevated expression levels in sedentary and exercised GK groups. Moreover, the expression levels of genes in group 2 were higher only in exercised GK rats.

### Functional enrichment analysis of DEGs

**Figure 2:**
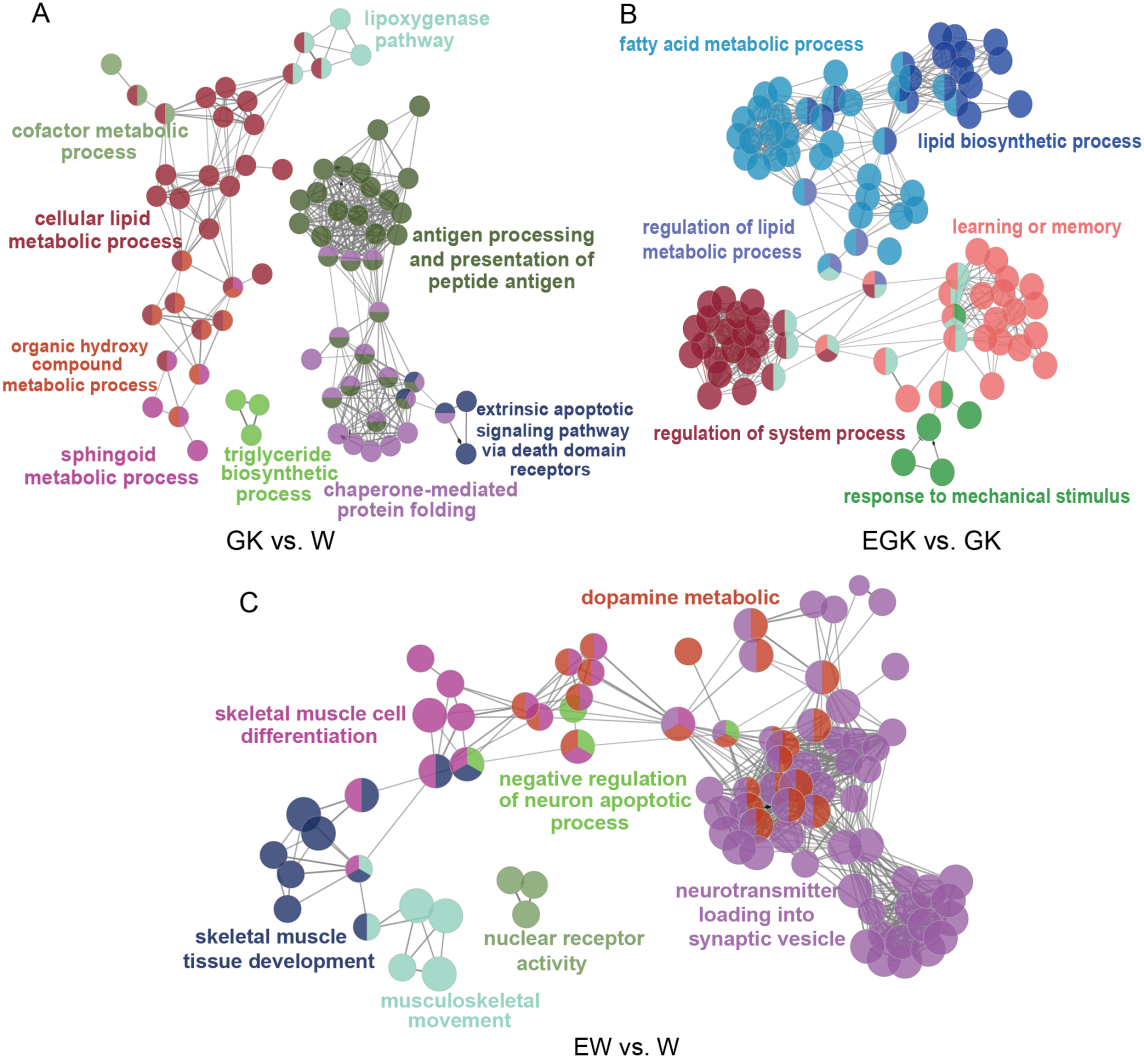
Networks based on significantly enriched GO terms in the three comparisons. DEGs were analyzed using plug-in ClueGO of Cytoscape to determine significantly enriched GO terms (FDR < 0.05). Functionally grouped network with GO terms as nodes linked based on their kappa score (≥ 0.3), where only the most significant term per group is presented. Networks with at least 3 GO terms are presented. The network of GO terms in (A) GK vs. W, (B) EGK vs. GK, (C) EW vs. W.

**Figure 3:**
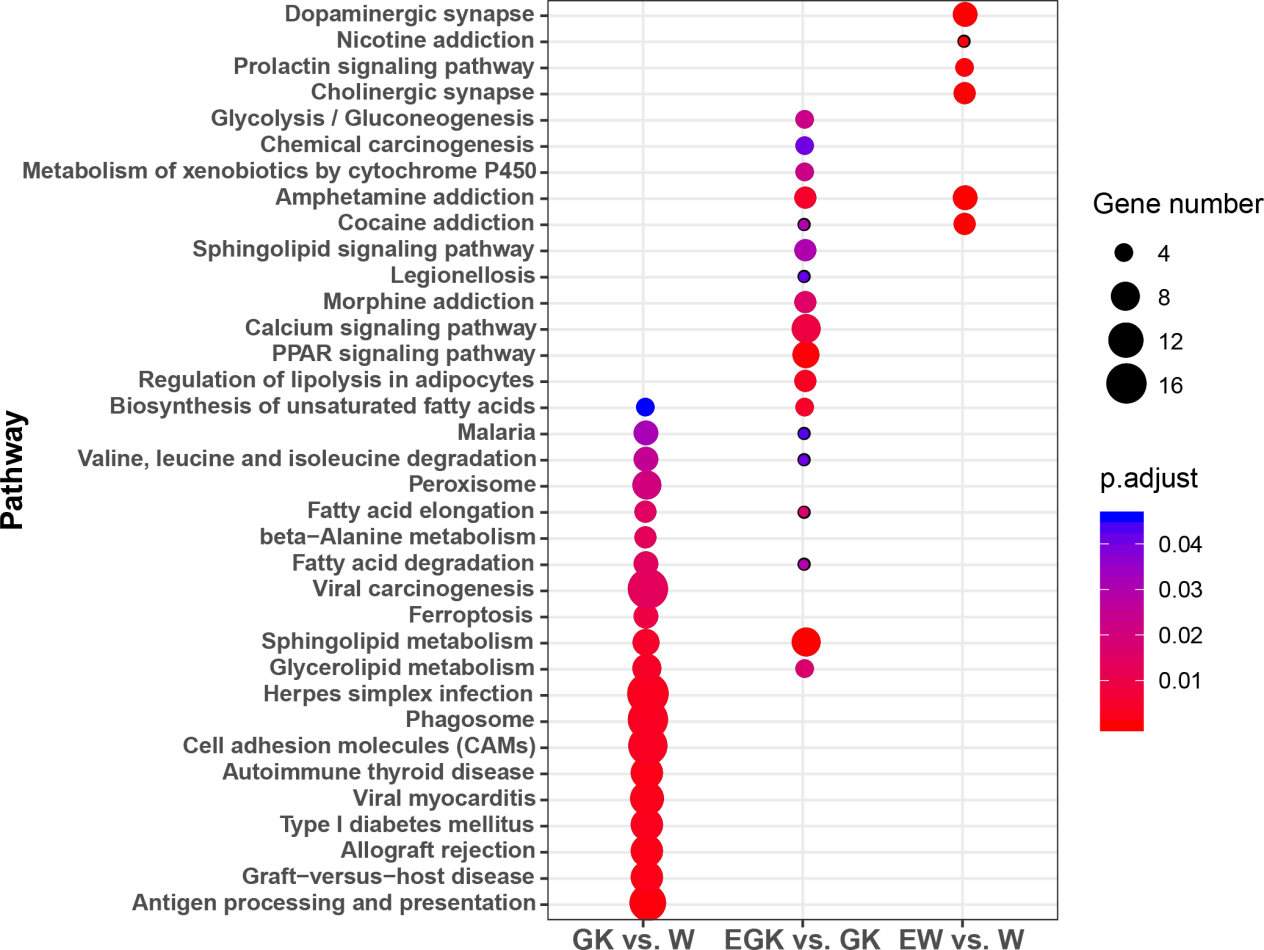
Significantly enriched KEGG pathways in the three comparisons. The color and size of the points were scaled with respect to adjusted *p*-values and the number of related DEGs, respectively.

To explore the potential functions of DEGs in the three comparisons, we performed GO and KEGG pathway enrichment analysis by GlueGO plug-in of Cytoscape. For the GO classification, the DEGs were divided into three ontologies: biological processes (BP), cellular compartments (CC), and molecular functions (MF). The differential expression of 726 genes between GK and Wistar rats were enriched in 124 GO terms, of which 96, 6, and 22 terms were enriched in BP, CC, and MF, respectively (Table S4). These GO terms were further clustered into nine groups and visualized using ClueGO (Fig. 2A). These groups included antigen processing and presentation of peptide antigen, cellular lipid metabolic process, chaperone-mediated protein folding, sphingoid metabolic process, triglyceride biosynthetic process, and so on, indicating that these enriched GO terms were primarily related to the immune system and lipid metabolism. Moreover, we found significant enrichment of 31 KEGG pathways (Table S4). Several pathways were also related to lipid metabolism, including glycerolipid metabolism, sphingolipid metabolism, fatty acid degradation, fatty acid elongation, and biosynthesis of unsaturated fatty acids (Fig. 3). These observations suggested that the metabolism of lipid might be dysfunctional in the hypothalamus of GK rats.

For the exercised GK vs. GK comparison, 197 DEGs were enriched at significant levels in 448 GO terms and 18 KEGG pathways (Table S4). Among these GO terms, 376 were enriched in BP, 34 were enriched in CC, and 38 were enriched in MF. After clustering, the GO terms were mainly related to fatty acid metabolic process, lipid biosynthetic process, regulation of lipid metabolic process, learning or memory, regulation of system process, and response to mechanical stimulus (Fig. 2B). The top five significantly enriched KEGG pathways were sphingolipid metabolism, PPAR signaling pathway, regulation of lipolysis in adipocytes, biosynthesis of unsaturated fatty acid, and amphetamine addiction (Table S4). Moreover, we found four pathways (glycerolipid metabolism, sphingolipid metabolism, fatty acid elongation, and fatty acid degradation) related to lipid metabolism were enriched in both comparisons including GK vs. Wistar and exercised GK vs. GK (Fig. 3). These findings suggested that acute exercise affected lipid metabolic processes, regulation of system process, and nervous system in the GK rats, which might contribute to the improvement of hyperglycemia and insulin sensitivity.

To identify the effects of acute exercise in the Wistar rats, we also performed functional enrichment analysis using the 98 genes whose expression was significantly changed in the exercised Wistar rats in comparison with the controls. We identified 93 GO terms, of which 70 were enriched in BP, four were enriched in CC, and 19 were enriched in MF (Table S4). The GO terms were mainly clustered into the neurotransmitter loading into synaptic vesicle and dopamine metabolic process, which were related to the nervous system (Fig. 2C). We also found that many GO terms were clustered into skeletal muscle tissue development and skeletal muscle cell differentiation, which might be caused by exercise stimulus. The KEGG pathway enrichment analysis showed that six pathways were significantly enriched, containing amphetamine addiction, cocaine addiction, dopaminergic synapse, cholinergic synapse, prolactin signaling pathway, and nicotine addiction (Table S4, Fig. 3). The pathways of amphetamine addiction and cocaine addiction were also enriched in the exercised GK vs. GK comparison. The functional enrichment analysis of DEGs in the exercised Wistar revealed that the nervous system might be influenced by exercise, and the observation was consistent with that observed in the exercised GK.

### Genes associated with T2D and related phenotypes

**Figure 4:**
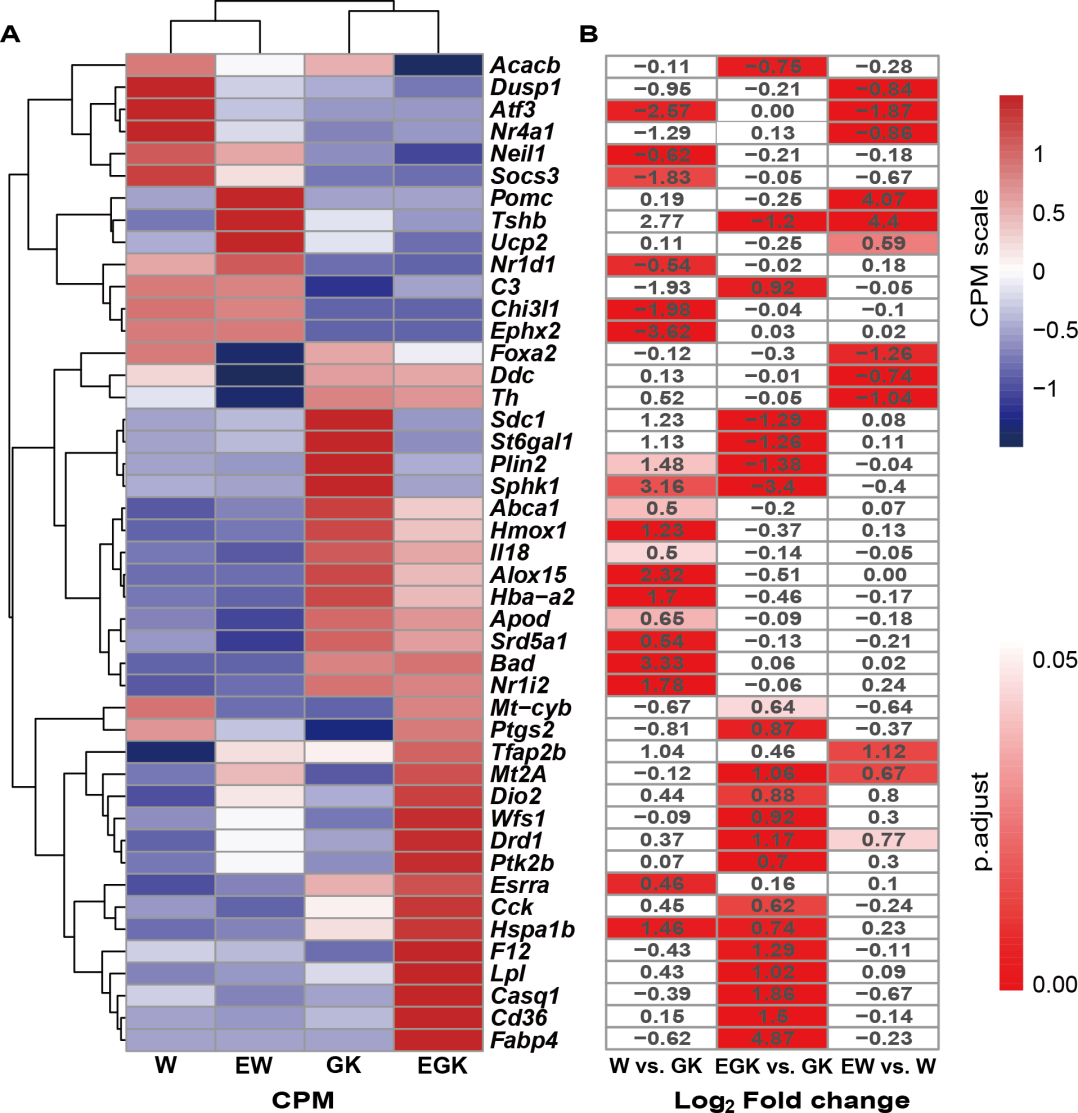
The DEGs associated with T2D and related phenotypes. (A) The heatmap for expression of these DEGs in the four groups (W, EW, GK, EGK). (B) The log_2_ fold change and FDR of these DEGs in the three comparisons. The red rectangle indicates the gene with differential expression. The number in each rectangle indicates the value of log_2_ fold change.

Among the 957 genes whose expression was significantly changed in the hypothalamus of three comparisons, 28 genes were annotated in the Rat Genome Database (RGD) as associated with T2D (Table S5). According to phenotype annotations in the RGD, three, seven, 25 and 23 DEGs were related to glucose intolerance, hyperglycemia, hyperinsulinemia, and insulin resistance, respectively (Table S5). Altogether, we found 45 DEGs referred to in the RGD that associated with T2D and multiple related phenotypes. The log_2_ fold change, scaled expression levels, and adjusted *p*-values of these DEGs were presented in Figs. 4A and 4B. Among these DEGs, *Plin2*, *Alox15*, *Nr1i2*, and *Nr1d1* involved in lipid metabolism-related GO terms and KEGG pathways were found in the GK rats. After exercise, the expression of *Plin2* was reversed in the GK rats. Moreover, the expression levels of *Cd36* and *Lpl* associated with lipid sensing in the hypothalamus were significantly upregulated in the exercised GK rats. Two other upregulated genes *Wfs1* and *Cck,* which involved in the regulation of the system process and nervous system, were associated with T2D and insulin resistance, respectively. In the exercised Wistar rats, the expression levels of *Pomc* and *Th* genes were significantly changed, which might affect appetite by regulating the nervous system.

### RNA-Seq data validation

**Figure 5:**
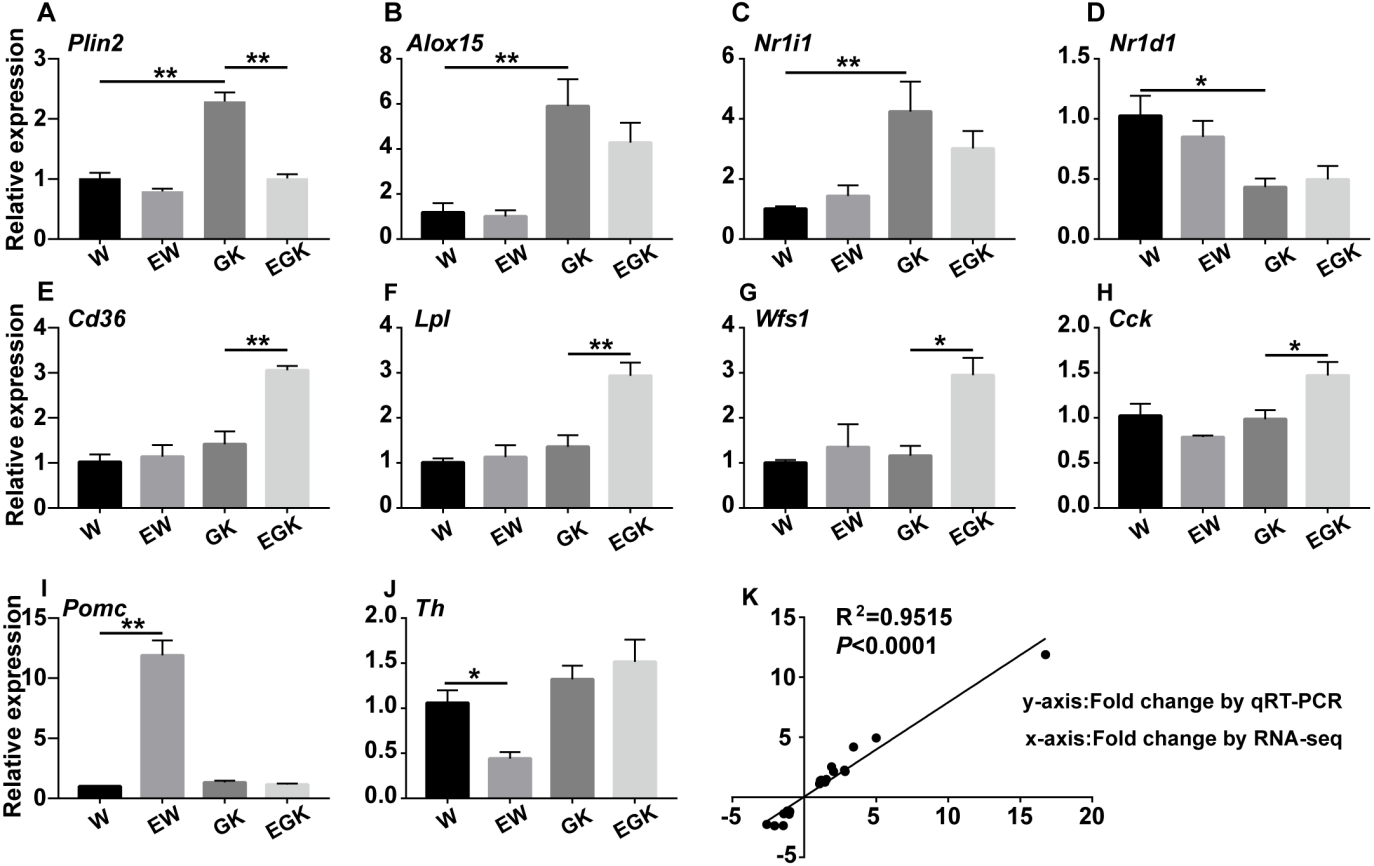
The relative expression levels measured by qRT-PCR. (A–J) qRT-PCR results of selected genes (*Plin2*, *Alox15*, *Nr1i2*, *Nr1d1*, *Cd36*, *Lpl*, *Wfs1*, *Cck*, *Pomc*, and *Th*) in the four groups. Transcript levels were normalized to host gene *Actb*. Analyses of data were done by ANOVA, and differences between groups were identified by Tukey’s *post hoc* analysis. Values are presented mean ± SEM, *n* = 6/group; **: *p* < 0.01, *: *p* < 0.05. (K) Correlation of the relative expression levels of selected genes determined by RNA-Seq and qRT-PCR in the four groups. The *x*-axis represents the RNA-Seq data, and the *y*-axis represents the qRT-PCR data. The *R*^2^ value and *p*-value are shown.

To validate the reproducibility and accuracy of the RNA-Seq data, 10 DEGs (*Plin2*, *Alox15, Nr1i2*, *Nr1d1*, *Cd36*, *Lpl*, *Wfs1*, *Cck*, *Pomc*, and *Th*) associated with T2D or exercise were chosen for transcript analysis by qRT-PCR (Figs. 5A–5J). The results exhibited similar patterns like that of RNA-Seq, with a correlation coefficient of 0.95 (*p* < 0.0001), indicating that our RNA-Seq data were accurate and reliable (Fig. 5K).

## Discussion

Physical activity plays a vital role in T2D prevention and treatment, and the molecular mechanisms associated with the beneficial effects have been investigated for many years (Accattato et al., 2017; La Gerche et al., 2015; Colberg et al., 2010). However, these studies mainly focused on the peripheral tissues, so that the contributions of the central nervous system are relatively neglected. The hypothalamus is a vital region of the central nervous and plays a crucial role in regulating energy balance (Lopez et al., 2016). Hypothalamic abnormalities were previously found to correlate with T2D development (Jais & Bruning, 2017). To explore the hypothalamic adaptive mechanisms after exercise, we assessed the transcriptome of the hypothalamus in the type 2 diabetic GK rats and control Wistar rats with or without acute exercise using RNA-Seq technology. DEGs analysis revealed that 726 genes were significantly altered in GK rats compared with Wistar rats. Of these DEGs, 507 genes were also identified in our previous study of the hypothalamic transcriptome in GK rats (Meng et al., 2016). The partial differences of GK rat transcriptomic profiles in two studies might be related to the change in the breeding environment (Portha et al., 2012). At 2 h post-exercise, 197 and 98 DEGs were observed between exercised GK and GK, and exercised Wistar and Wistar rats, respectively. However, we did not identify genes that were uniquely responsive to exercise in both exercised GK and Wistar rats. GO and KEGG enrichment analysis and RGD database annotations were performed on these DEGs to detect molecular mechanisms associated with T2D or stimulated by acute exercise in the hypothalamus.

For the comparison of GK vs. Wistar, multiple GO terms and KEGG pathways related to lipid metabolism were significantly enriched, including the fatty acid, sphingolipid, triglyceride, and glycerolipid metabolic processes. Many studies have demonstrated that the disorder of lipid metabolism in the hypothalamus could cause hyperglycemia and insulin resistance, leading to the development of T2D (Yue & Lam, 2012; Lam et al., 2005; Bruce, Zsombok & Eckel, 2017). Among these DEGs, the upregulated expression of *Plin2*, *Alox15*, and *Nr1i2*, and the downregulated expression of *Nr1d1* might correlate to hypothalamic disorders of lipid metabolism and contribute to hyperglycemia or insulin resistance in type 2 diabetic GK rats according to RGD annotations and previous studies. The gene *Plin2* encodes Perilipin-2 (PLIN2) which has a vital function as an LD-coating protein in the formation of intracellular lipid droplets (LDs). Previous studies reported that muscle PLIN2 protein expression was significantly increased in Zucker diabetic fatty rats during the development of T2D and negatively correlated to insulin sensitivity in T2D patients (Minnaard et al., 2009). Transgenic studies found that overexpression of *Plin2* inhibited glucose uptake stimulated by insulin in cells, whereas a whole-body knockdown of *Plin2* enhanced insulin sensitivity and glucose tolerance in diabetic mice (Senthivinayagam et al., 2013; Cho & Kang, 2015; Chang et al., 2010). In light of these, the elevated expression of *Plin2* in the hypothalamus might contribute to insulin resistance of GK rats. After acute exercise, the expression of *Plin2* was decreased significantly, which might lead to an improvement in insulin sensitivity and hyperglycemia in the exercised GK rats. In addition, *Alox15* encodes 12/15-lipoxygenase (12-LOX, 15-LOX) which is linked to fatty acids metabolism and is involved in the pathogenesis of T2D (Imai et al., 2013; Chakrabarti et al., 2009). The upregulated *Alox15* in the adipose impaired insulin signaling, but the whole-body and adipose-specific deficiency of *Alox15* attenuated hyperglycemia and insulin resistance in mice (Cole et al., 2012; Sears et al., 2009; Chakrabarti et al., 2009). In the GK rats, the significantly upregulated expression of *Alox15* in the hypothalamus might be implicated in elevated blood glucose and insulin resistance. Pregnane X receptor (PXR), encoding by *Nr1i2,* impacted energy homeostasis by regulating multiple metabolic pathways (Gao & Xie, 2010). Ablation of *Nr1i2* in the whole-body could protect mice from hyperglycemia and insulin resistance and showed improvement of metabolic functions and insulin sensitivity in T2D (He et al., 2013; Spruiell et al., 2014). Also, the transgenic activation of *Nr1i2* exacerbated insulin resistance and glucose intolerance in mice (He et al., 2013). These studies suggested that significant upregulation of *Nr1i2* in the hypothalamus might be associated with insulin resistance and hyperglycemia in the GK rats. NR1D1, also known as Rev-erbα, encoding by *Nr1d1*, has been shown to control lipid and glucose metabolism and correlate to T2D (Zhang et al., 2015; Zhang et al., 2013). Whole-body knockout of *Nr1d1* affected energy utilization and elevated plasma glucose level in mice (Delezie et al., 2012). Thus, decreased expression of *Nr1d1* in the hypothalamus might be linked to hyperglycemia in the GK rat.

For the comparison of exercised GK vs. GK, GO and KEGG enrichment analysis demonstrated an overrepresentation of DEGs related to lipid metabolism, including fatty acid metabolic process, regulation of lipid metabolic process, and lipid biosynthetic process, which gave similar results in the GK when compared with Wistar rats. It can be speculated that exercise exerted a beneficial influence on lipid metabolism which might be dysfunctional in the diabetic GK rats, such as the reversed transcriptional activity of *Plin2* after exercise as discussed above. The DEGs were also enriched in GO terms related to the nervous system regulation. Considering RGD annotations and previous research, the downregulated *Plin2* and upregulated *Cd36*, *Lpl*, *Wfs1*, and *Cck* might improve lipid metabolism or neuronal responsiveness to hyperglycemia in the hypothalamus, leading to the amelioration of hyperglycemia and insulin sensitivity in the exercised GK rats. CD36, a fatty acid translocase, encoding by *Cd36*, is associated with long-chain fatty acid transport and plays an essential role in the metabolism of fatty acid and insulin action (Koonen et al., 2010; Duca & Yue, 2014). Several lines of evidence supported its prominence as a regulator of hypothalamic lipid sensing and glucose balance. Le Foll et al. found that CD36 accounted for more than 50% fatty acid sensing in ventromedial hypothalamic neurons (Le Foll et al., 2009). The knockdown of *Cd36* in the hypothalamus led to markedly higher glucose and insulin levels, as well as significant glucose intolerance (Le Foll et al., 2013; Le Foll, Dunn-Meynell & Levin, 2015). The *Lpl* gene encodes lipoprotein lipase (LPL) which can hydrolyze triacylglycerol-enriched lipid particles to provide lipid signals for modulating body weight and energy homeostasis in the central nervous system (Wang et al., 2011). In the hypothalamus, the deficiency of *Lpl* caused higher blood glucose and insulin levels, and overexpression of *Lpl* led to increased resting metabolic rate (Laperrousaz et al., 2017). Thereby, the expression levels of *Cd36* and *Lpl* were significantly increased in the hypothalamus after acute exercise, which might enhance lipid sensing to improve T2D and related phenotypes in the GK rats. Besides, the *Wfs1* gene encodes a transmembrane protein called wolframin (WFS1), which is involved in the regulation of Ca^2+^ concentrations in the endoplasmic reticulum (ER). Impaired glucose tolerance in the *Wfs1*-deficient mice has been described by several studies (Ishihara et al., 2004; Koks et al., 2009). Furthermore, overexpression of *Wfs1* in cells increased ER Ca^2+^ levels to enhance neuronal responsiveness to hyperglycemia (Ando et al., 2018; Takei et al., 2006). Hence, the upregulated *Wfs1* in the hypothalamus might be associated with improvement of hyperglycemia in the exercised GK rats. *Cck* encodes cholecystokinin (CCK), a neuropeptide that is abundantly distributed in the hypothalamus. The injection of CCK into the hypothalamus showed improved insulin sensitivity as well as glucose tolerance in rats (Zhu et al., 2012). The increased expression of *Cck* in the hypothalamus might contribute to ameliorating hyperglycemia in the exercised GK group.

The hypothalamus regulates whole-body energy homeostatic functions including control of food intake via two distinct neuronal populations: the first set of neurons express anorexigenic peptide POMC, and the other set express neuropeptide Y (NPY) and orexigenic peptides agouti-related protein (AgRP) (Jeong, Kim & Lee, 2014). Recent studies have shown that both acute and chronic exercise suppressed hyperphagia via modulating the expression of *Npy* and *Pomc* in the hypothalamus (Ropelle et al., 2010). CCK was involved in the regulation of appetite for reducing feed intake when injected into the hypothalamus (Blevins, Stanley & Reidelberger, 2000). Genetic and pharmacological studies have focused on how CCK altered the behavior of feeding, and the results indicated that it might rely on activation of anorexigenic POMC neurons or suppression of orexigenic NPY signaling to inhibit food intake (Fan et al., 2004; Chen et al., 2008). However, we observed no significant change in *Npy* and *Pomc* expression levels in the exercised GK rats. Therefore, it is difficult to determine whether appetite was affected by upregulated *Cck* in the exercised GK rats or not. In the hypothalamus of exercised Wistar rats, the expression of *Pomc* was found significantly increased, suggesting that exercise could inhibit feed intake in the Wistar rats, which was in agreement with the previous study (Cavalcanti-de-Albuquerque et al., 2018).

Oxidative stress, a common phenomenon, was linked to physical exercise (Powers, Nelson & Hudson, 2011). It has been reported that only one acute physical exercise can induce oxidative stress and activate antioxidant protection mechanisms in sedentary individuals (Accattato et al., 2017). These responses to the acute exercise might be associated with increased productions of reactive oxygen species and altered the expression of related genes responding to oxidative stress (Powers, Nelson & Hudson, 2011; Muthusamy et al., 2012). In this study, we considered whether oxidative stress occurred after acute exercise based on transcriptome changes. However, we did not find any GO terms and KEGG pathways related to oxidative stress were significantly enriched in both exercised GK and Wistar groups. This result may be due to the tissue specificity of the hypothalamus and the intensity of exercise done in our study (Kim, Kim & Son, 2018).

Earlier studies demonstrated that transcriptional responses changed at different time points after exercise (Louis et al., 2007; Dickinson et al., 2018). In the current study, we chose the time point (2 h post-exercise) based on previous studies on acute exercise (Pourteymour et al., 2017; Takimoto & Hamada, 2014), and identified some key DEGs which might contribute to the improvement of hyperglycemia and insulin sensitivity in the exercised GK rats. However, this study does not adequately reflect all the important changes in the transcriptome of hypothalamus after acute exercise and has ignored immediate gene expression changes in response to exercise. Hence, more time points need to be considered for future research.

In addition, to validate the results of RNA-Seq, several key DEGs were selected for transcript analysis by qRT-PCR using the sequenced samples. The results showed that the RNA-Seq data were reliable and accurate. However, the use of the same samples for both RNA-Seq and qRT-PCR studies reduced the overall robustness of the findings, and a new set of samples are needed to replicate the mRNA findings in future studies.

## Conclusion

Multiple genes were identified differentially expressed among the three comparisons through analysis of transcriptional profiling of the hypothalamus in GK and Wistar with or without acute exercise. Functional enrichment analysis revealed that the DEGs were involved in lipid metabolism-related GO terms and KEGG pathways in both GK and exercised GK rats. Moreover, DEGs involved in the nervous system related GO terms and KEGG pathways were identified in both exercised GK and Wistar rats. According to the RGD annotations, 45 DEGs were associated with T2D and related phenotypes. Based on previous studies, we found the dysregulations of *Plin2*, *Alox15*, *Nr1i2*, and *Nr1d1* genes related to lipid metabolism might implicate to the disorders of the hypothalamus in the diabetic GK rats and contribute to the hyperglycemia in them. In the exercised GK rats, the expression of *Plin2* was decreased, and that of *Cd36* and *Lpl* genes enhancing lipid sensing were significantly upregulated, which might improve lipid metabolism in the hypothalamus, consequently ameliorating T2D. Moreover, we found that the expression of *Wfs1* and *Cck* related to the nervous system was increased, which might contribute to the beneficial effects of acute exercise on T2D. Taken together, our study provided an understanding of the transcriptional processes stimulated by acute exercise and in-depth exploration of these changes in the type 2 diabetic GK rats, which might help in strategies to prevent and treat T2D.

##  Supplemental Information

10.7717/peerj.7743/supp-1 Table S1 The information about the sequence reads and genome alignment

10.7717/peerj.7743/supp-2 Table S2 The primers for qRT-PCR

10.7717/peerj.7743/supp-3 Table S3 The DEGs and their details

10.7717/peerj.7743/supp-4 Table S4 The significantly enriched GO terms and KEGG pathways

10.7717/peerj.7743/supp-5 Table S5 The DEGs referred to in RGD as associated with T2D and related phenotypes

## Additional Information and Declarations

### Data Availability

The following information was supplied regarding data availability: The raw data are available at NCBI SRA database (accession number: PRJNA505092).
